# Brain mechanisms underlying the influence of emotions on spatial decision-making: An EEG study

**DOI:** 10.3389/fnins.2022.989988

**Published:** 2022-09-30

**Authors:** Yanyan Zhao, Danli Wang, Xinyuan Wang, Steve C. Chiu

**Affiliations:** ^1^State Key Laboratory for Management and Control of Complex Systems, Institute of Automation, Chinese Academy of Sciences, Beijing, China; ^2^School of Artificial Intelligence, University of Chinese Academy of Sciences, Beijing, China; ^3^ECE Department, Idaho State University, Pocatello, ID, United States

**Keywords:** emotion, decision-making, spatial task, electroencephalography (EEG), microstate analysis, effective connectivity analysis, graph theoretical analysis, event-related potentials (ERP)

## Abstract

It is common for people to make bad decisions because of their emotions in life. When these decisions are important, such as aeronautical decisions and driving decisions, the mistakes of decisions can cause irreversible damage. Therefore, it is important to explore how emotions influence decision-making, so as to avoid the negative influence of emotions on decision-making as much as possible. Although existing researchers have found some mechanisms of emotion's influence on decision-making, only a few studies focused on the influence of emotions on decision-making based on electroencephalography (EEG). In addition, most of them were focused on risky and uncertain decision-making. We designed a novel experimental task to explore the influence of emotion on spatial decision-making and recorded subjective data, decision-making behavioral data, and EEG data. By analyzing these data, we came to three conclusions. Firstly, we observed three similar event-related potentials (ERP) microstates in the decision-making process under different emotions by microstate analysis. Additionally, the prefrontal, parietal and occipital lobes played key roles in decision-making. Secondly, we found that the P2 component of the prefrontal lobe presented the influence of different emotions on decision-making by ERP analysis. Among them, positive emotion evoked the largest P2 amplitude compared to negative emotions and no stimuli. Thirdly, we found some graph metrics that were significantly associated with decision accuracy by effective connectivity analysis combined with graph theoretic analysis. In consequence, the finding of our study may shed more light on the brain mechanisms underlying the influence of emotions on spatial decision-making, thereby providing a basis for avoiding decision-making accidents caused by emotions and realizing better decision-making.

## Introduction

As a high-level cognitive process, decision-making involves extensive and complex human behavior (Neubert et al., 2015), and is the core of human cognitive information processing (Simon, 1978). Damasio pointed out that decision-makers needed knowledge in the following aspects: the context in which decisions were made, different response options, and the immediate or long-term results of different options (Damasio, 2006). In this process, decision-makers are supposed to calmly assess the potential consequences of their decisions and choose options that maximize the benefits of these consequences (Loewenstein and Lerner, 2003). Early studies of decision-making generally assumed that people were rational. But in the 1980s, this view was challenged (Vecchiato et al., 2014).

Hebert Simon believed that decision-making theory was incomplete until the mechanism of emotion's effect on decision-making was clarified (Simon, 1967, 1990). Damasio defined emotion as the sum of changes in body and brain states caused by brain systems responding to some specific perceptual content, either actually occurring or recalled, associated with a particular object or event (Damasio, 2006). He outlined the neuroanatomical and cognitive framework of the influence of emotions on decision-making and proposed the somatic marker hypothesis. He believed that emotional responses (somatic markers) produced by people in decision-making could influence the decision-making behavior of subjects in uncertain and complex situations (Tranel and Damasio, 1991). Lopes et al. also pointed out that adding emotion into the decision-making model could greatly improve the explanatory power of the decision-making model (Lopes, 1987; Lopes and Oden, 1999). Therefore, from the perspective of decision theory, the influence of emotion on decision-making is an important topic that cannot be ignored. From the perspective of practical decision-making, emotion can jeopardize decision-making relevance and cognitive functioning. A large range of strong negative emotional consequences may provoke a temporary impairment of the decision-making process, which may lead to flight casualties and medical accidents (Causse et al., 2013; Robertson and Long, 2018). Therefore, from the perspective of practical decision-making, it is also very important to study the influence of emotion on decision-making.

For the above reasons, related studies on the influence of emotion on decision-making have gradually increased and many important results have been achieved (Lerner et al., 2015). Most of the early studies were carried out by subjective and behavioral data of subjects. For example, Heilman et al. (2010) induced the emotions of fear and disgust, and found that cognitive reappraisal of these negative emotions reduced risk aversion. Similarly, Kugler et al. explored the influence of fear and anger on risk-taking behaviors, and the results showed that compared with anger, fear would increase the choice frequency of risk avoidance, while anger reduced the choice frequency of risk avoidance (Kugler et al., 2012).

Brain is the most advanced and important part of nervous system, and activity in some brain regions can represent not only emotional regulation but also complex decision-making processes (Seymour and Dolan, 2008). Since the above research based on subjective scoring relied too much on subjective feelings of subjects and was unable to clarify the brain mechanisms of the complex cognitive processes, some researchers focused on physiological signals such as EEG. Based on EEG, Balconi et al. (2018) applied the Iowa Game Task (IGT) to explore the difference between Parkinson's patients with or without pathological gambling behavior. The results showed that Parkinson's patients with pathological gambling behavior had worse task performance. Moreover, EEG signal analysis showed that the low frequency bands of their frontal lobe increased. Giustiniani et al. (2019) found that correct decision-making under uncertain conditions is regulated by an individual's emotion, ability to encode information and explore information. In addition, EEG signal analysis indicated that the right/left theta asymmetry may reflect the individual's motivation tendency. Garrido-Chaves et al. (2021) applied IGT decision task to analyze the difference in the influence of emotion on decision-making between males and females. The results showed that there were no significant gender-related differences in behavioral performance, but women were more sensitive to failure. In women, but not in men, the feedback-related negativity (FRN) component showed a greater amplitude for losses than for wins.

Although the above studies have found many important brain mechanisms for the influence of emotions on decision-making, they focused on risky and uncertain decision-making. Some studies pointed out that risk and ambiguity are two fundamental parameters of decision-making (Zheng et al., 2020). However, decision-making can also be made under the condition that the result of choice is completely predictable (Iribe-Burgos et al., 2022). Spatial decision is a kind of decisions with predictable selection results and an important part of daily life. In addition, spatial decision-making is also characterized by the use of past experience to guide future decisions. So spatial decision-making is a good way to understand how the brain describes this experience and lays the foundation for reinforcement learning theory (Simon and Daw, 2011). More importantly, spatial decision-making is an indispensable part of driving decisions and aeronautical decisions (Stokes et al., 2014). When a driver or pilot is in the process of driving, he or she may be affected by emotions and make a bad decision, which may bring irreparable damage. Therefore, it is important to further explore the influence of emotions on spatial decision-making so as to avoid the negative influence of emotions on decision-making.

Recently, As goal-oriented decision research, spatial decision-making has attracted the attention of many researchers. Kaplan et al. used functional magnetic resonance imaging (fMRI) to explore the specific brain region of the prefrontal lobe in spatial decision-making. The interaction between the rostrodorsal medial prefrontal cortex (rd-mPFC) and the hippocampus was found to increase when deliberate planning was required, especially when planning led to accurate choice (Kaplan et al., 2017). Similarly, Epstein pointed out that hippocampal and entorhinal spatial codes were used in conjunction with frontal lobe mechanisms to plan routes during navigation (Epstein et al., 2017). A number of studies have also shown that spatial long-term memory and contextual memory depend on the Retrosplenial Cortex (RSC) (Corcoran et al., 2011; Katche et al., 2013; Cowansage et al., 2014; Milczarek et al., 2018; de Sousa et al., 2019), and damage to the RSC could impair navigation in both humans and rodents (Vann and Aggleton, 2002; Ino et al., 2007; Pothuizen et al., 2010). On this basis, Miller et al. trained rats with a continuous T-maze alternation task. They found that the RSC developed a distributed population-level representation of the rat's spatial location and current trajectory to the goal as the rats learned (Miller et al., 2019). Although many key brain mechanisms have been found in these studies, the role of emotion in spatial decision-making remains unclear and needs further investigation. In addition, most spatial decision-making studies are fMRI studies, which have low temporal resolution. EEG possesses high temporal resolution with about 1 ms, allowing researchers to study phase changes in response to decision tasks, which can effectively compensate for this problem. Furthermore, the sufficient time resolution enables the capture of macroscopic dynamics of brain activation and synchronization (Delorme et al., 2002; da Silva, 2013; Alarcao and Fonseca, 2017).

For existing studies, although they has found some brain mechanisms of the influence of emotions on decision-making, there are still the following problems: **(P1)** There are few studies focusing on the influence of emotions on decision-making based on EEG, and signal analysis methods are simple (Davis et al., 2011; Lanini-Maggi et al., 2014; Takács et al., 2015). Most of them focus on some common time-domain and frequency-domain features, such as ERP and band power. **(P2)** Conclusions obtained by different research methods are not uniform. For example, Charpentier et al. (2017) showed that fear would reduce risk propensity, which contradicts Heilman et al.'s finding that fear would increase risk propensity. By summarizing a large number of literature on the impact of fear on risk tasks, Wake et al. (2020) further found that the conclusions of different studies were different. **(P3)** The existing research on the influence of emotions on decision-making mainly uses risky decision tasks or uncertain decision tasks. Most of the research on spatial decision-making focuses on the decision task itself and ignores the influence of emotions on it. Most of them are fMRI studies. As a result, The influence of emotions on spatial decision-making has rarely been explored with EEG.

In order to solve the above problems and make up for the lack of research on the influence of emotions on spatial decision-making, we designed a novel experimental task based on EEG in this paper. In the selection of emotions, we refer to the research of Zheng et al. (2018), Gao and Maurer (2009), in which one kind of positive emotion and two kinds of negative emotions (fear, sad) are specifically analyzed. In addition, there are many studies exploring the influence of sad and fear emotions on risky or economic decision-making (Harlé et al., 2012; Wake et al., 2020). As a result, we choose these three emotions. During the experiment, we recorded subjective data, decision-making behavioral data, and EEG data of the subjects. The results of the matched samples *t*-test showed that emotions were effectively stimulated. Then, similar microstates of the brain under different emotions in the decision-making process were obtained by microstate analysis, and key brain regions of the decision task were found. Focused on the key brain regions, ERP analysis was used to explore the role of different emotions in the decision-making process. Finally, by exploring the correlation between decision behavioral data and graph metrics of the brain network, the metrics and corresponding key regions in key frequency bands that significantly affected decision accuracy were found.

## Materials and methods

### Experimental design

#### Hypotheses

The current study has the following hypotheses: **(H1)** subjects' emotions are effectively stimulated by emotional films. **(H2)** The ERP microstates of the subjects under different emotions show similar patterns in decision-making task. **(H3)** The stimulated emotions have an impact on the decision-making process and can be reflected in the EEG characteristics during the task. **(H4)** There is a correlation between decision accuracy and graph metrics of brain network.

#### Subjects

Sixteen Chinese postgraduate students in University of Chinese Academy of Sciences participated in this experiment. Their ages ranged from 22 to 28 years old (Mean= 23.25, std = 1.57, 11 females, 5 males). All of them had normal vision or corrected-to-normal vision. None reported any known neurological or psychiatric disease. And they were required to get a good rest before the experiment. All subjects gave their written informed consent and were paid for their participation.

#### Emotional stimuli

**Figure 1:**
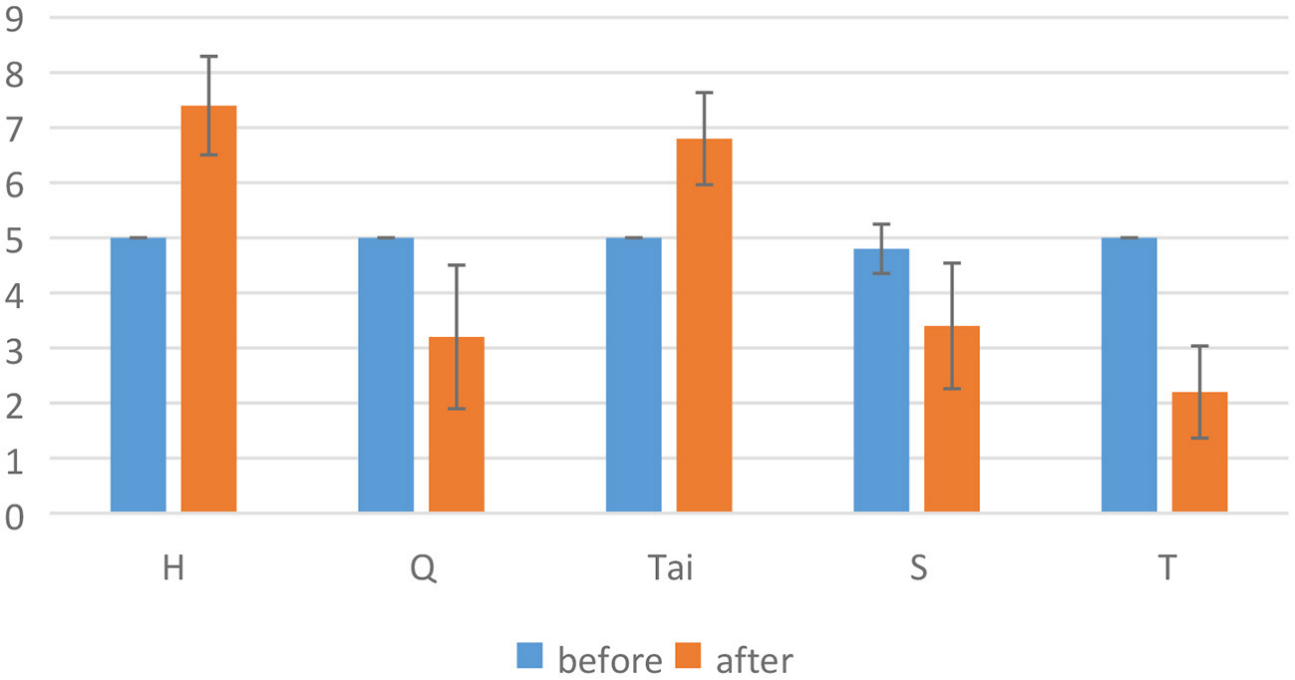
Valence score of preliminary experimental evaluation.

In the experiment, video clips were used to stimulate emotions (Gross and Levenson, 1995; Alarcao and Fonseca, 2017). In order to select emotional stimuli, we conducted a preliminary experimental evaluation. In the preliminary experimental evaluation, we selected Aftershock clip (T) (Zheng and Lu, 2015), Lost in Thailand clip (Tai) (Zheng and Lu, 2015), Bean clip (H) (Soleymani et al., 2011), The Shining clip (S) (Schaefer et al., 2010) and Curve clip (Q). The first four referred to the emotional stimuli of public datasets, and the last was an online classic fear clip. Of the five films, Bean and Lost in Thailand clips were used to stimulate positive emotions, while the rest were used to stimulate negative emotions. In negative emotions, The Shining and Curve clips were used to stimulate fear and Aftershock clip was used to stimulate sad. To ensure the effect of emotional stimuli, we asked five subjects to evaluate these five films. The figure of valence from preliminary experimental evaluation were shown in Figure 1. The results showed that Bean, Curve and Aftershock clips can more effectively stimulate positive and negative emotions (the valence has a larger difference before and after stimulating). As a result, we choose these three emotional films. Among them, Bean clip lasts 3 min 17 s, the Curve clip lasts 3 min 9 s, and the Aftershock clip lasts 3 min 29 s.

#### Decision task

The decision task of this experiment is based on the spatial decision task proposed by Kaplan et al. (2017). In this task, we used a software called Blender (http://www.blender.org) to generate maze map.

**Figure 2:**
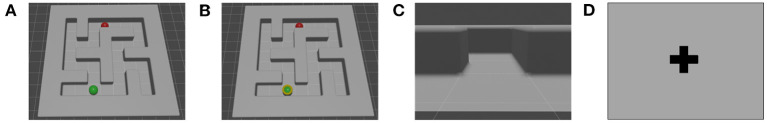
Spatial decision task. **(A)** Picture 1. In this picture, green ball is the starting point and red ball is the ending point. Subjects need to infer the shortest path from the starting point to the ending point. **(B)** Picture 2. Picture 2 adds a choice point (yellow circle) on the intersection of the shortest path based on Picture 1. At this point, subjects need to judge which direction to take is the shortest path. **(C)** Picture 3. Picture 3 featured a first-person viewpoint of the choice point. Subjects should operate in this picture (W: go forward, A: go left, D: go right, S: all directions have the same path length). **(D)** Interstimulus interval (ISI), during which the subjects were allowed to rest.

As shown in Figures 2A–C, in each trial, the task shows three different states of a maze in sequence. Figure 2A is a three-dimensional maze, in which the green ball is the starting point and the red ball is the ending point. Subjects need to infer the shortest path from the starting point to the ending point. Figure 2B adds a choice point (yellow circle) on the intersection of the shortest path based on Figure 2A. At this point, subjects need to judge which direction to take is the shortest path. Figure 2C featured a first-person viewpoint of the choice point. Subjects should operate in this maze (W: go forward, A: go left, D: go right, S: all directions have the same path length). Figure 2D is the interstimulus interval (ISI), during which the subjects were allowed to rest. After preliminary experimental evaluation and adjustment, Figure 2A displays 3,000 ms, Figure 2B displays 500 ms, Figure 2C displays 1,500 ms, ISI displays 1500 ms. As a result, the decision task duration of each trial is 6.5 s.

#### Subjective measures

**Figure 3:**
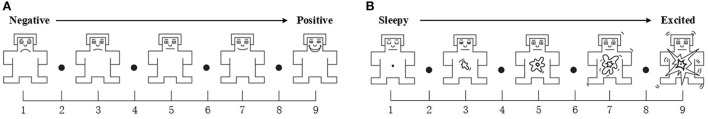
V-A scale. **(A)** Valence, It goes from very negative feelings to very positive. **(B)** Arousal, It goes from sleepy to excited.

Before and after each emotional stimulus, the subjects were asked to fill out V-A scale (Russell, 1980). The V-A scale is shown in Figure 3. It ranges from 1 to 9. For valence, the score from low to high means negative to positive. For arousal, the score from low to high means sleepy to excited.

### Procedure

**Figure 4:**
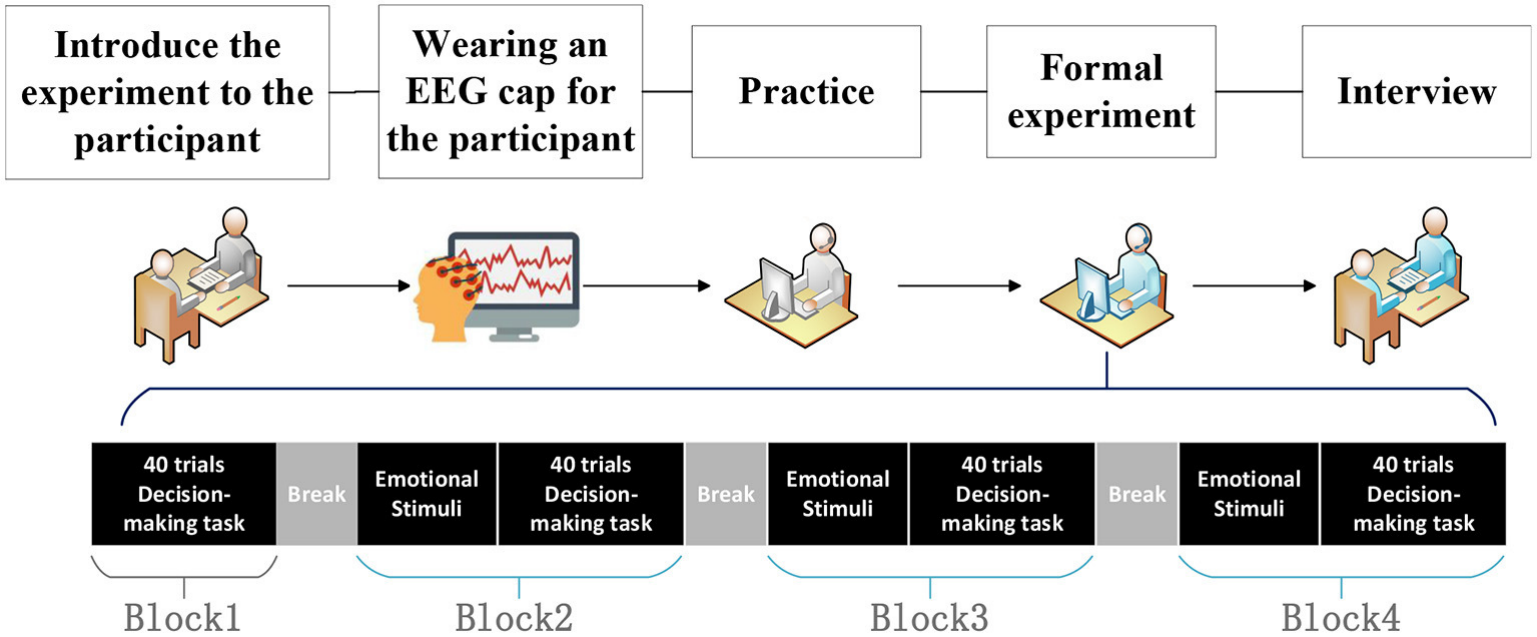
Experimental procedure. At first, we introduced our experiment to subjects. Then, they were asked to wear an EEG cap to collect data. After this, they practiced the decision task. In the formal experiment, the subjects were asked to complete four blocks of decision task. Finally, they had an interview.

Experimental procedure is shown in Figure 4. At first, we introduced our experimental procedure to subjects. Then, subjects were asked to wear an EEG cap to collect data. Afterward, subjects practiced decision task to become familiar with the corresponding operations. The practice task consists of 10 trials, containing all possible operations (W: go forward, A: go left, D: go right, S: all directions have the same path length). In the formal experiment, the subjects were asked to complete four blocks of decision task. Each block contains 40 trials, which means the decision task duration of each block is 4 min 20 s. Block1 contains only spatial decision task, no emotional stimuli, which is denoted as None. Block2 to block4 are emotional stimuli first, followed by spatial decision task. Block2 to block4 is denoted as H, Q or T, depending on the emotional stimulus (H: Bean clip, Q: Curve clip, T: Aftershock clip). The duration of the formal experiment is about 30 min.

In order to avoid the interference of maze difficulty in the study of emotional influence on decision-making, we ensured four blocks contained the same number of mazes with the same difficulty, in which the difficulty of a maze was controlled by the number of maze paths, the length of maze paths, and the number of turning points. In order to avoid the influence of familiarity rather than emotional state on the decision task of the subjects, the sequence of emotional stimuli was disrupted. Every 6 subjects were subjected to emotional stimuli in the order of HQT, HTQ, TQH, THQ, QHT and QTH at block2, block3 and block4, respectively. For example, HQT means that block2, block3 and block4 use the emotional stimuli of Bean clip, Curve clip and Aftershock clip, respectively.

Finally, each subject had an interview. Through the interview, we further understood the emotional state, task feeling and task completion skills of the subjects.

#### Data analysis methods

**Figure 5:**
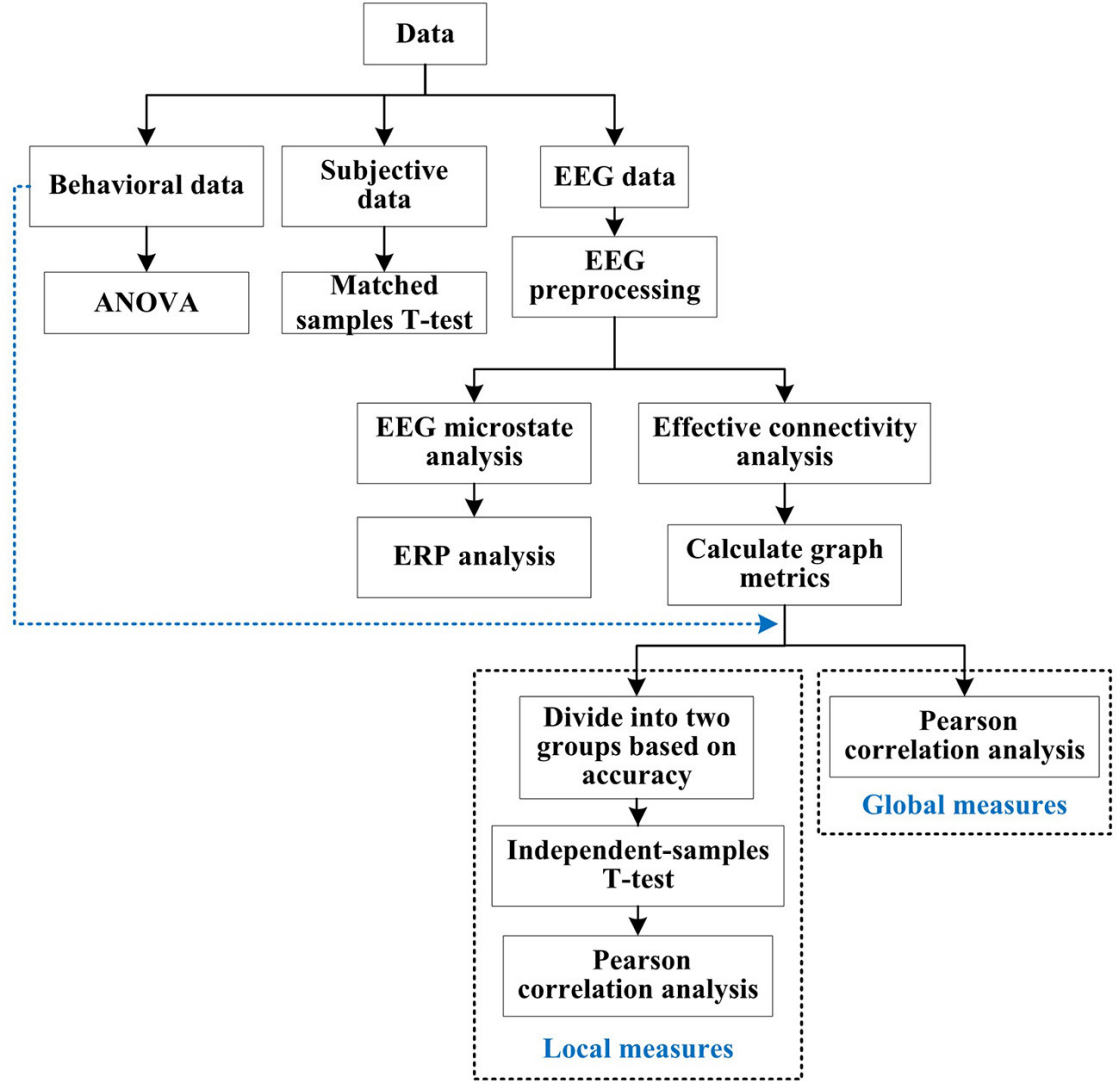
Data analysis process. Data were processed based on type, i.e. EEG, behavioral or subjective. Correlation between behavioral data and graph metrics (local and global measures) calculated by EEG data were analyzed in the final stage.

The data analysis process is shown in Figure 5. Subjective data were analyzed by matched samples *t*-test to verify the effect of emotional stimuli. Behavioral data were analyzed by analysis of variance (ANOVA) to explore the influence of emotions on accuracy and reaction time of decision task. For the EEG data, firstly preprocessed, and then used the microstate analysis to explore the similar microstates of decision task under different emotions, aiming to find the relevant brain regions of decision task. Secondly, according to the results of microstate analysis, ERP was used to analyze the difference in amplitude and latency of decision task related brain regions under different emotions, to determine the influence of emotions on the decision task. Finally, effective connectivity analysis combined with graph theoretic analysis was used to explore which graph metrics of the brain network corresponding to the electrode regions were significantly correlated with behavioral data.

##### Preprocessing

Due to the inevitable noise during EEG data acquisition, EEG signals are usually preprocessed before EEG data analysis. This study referred to the preprocessing method proposed by Yue et al. (2018), which mainly consisted of six steps: (1) EEG data was resampled to 250 Hz. (2) 1-40Hz band-pass filtering were used to remove high-frequency noise. Choosing 40 Hz as the upper bound of bandpass filtering can improve the signal-to-noise ratio and reduce the interference of high-frequency electromyography on EEG. On the other hand, it also retains the gamma component of 10 Hz width. ICA has a preference for low-frequency components of high-amplitude EEG, and selecting 1 Hz as the lower bound of band-pass filtering can correct ICA preference to a certain extent (Winkler et al., 2015). (3) Artifact Subspace Reconstruction (ASR) algorithm is used to remove noise and artifacts automatically. ASR automatically identifies the parts of the data without noise and artifacts to identify and eliminate the components with large variance and reconstruct the data from the remaining components. (4) Reconstruct deleted channel data by interpolation. (5) The average reference was set as the reference electrode. It has been proved that using the average reference would have better results (Hu et al., 2017; Wang et al., 2019b). (6) Independent Component Analysis (ICA), which is used to remove electroophthalmic artifacts.

##### Microstate analysis

Lehmann et al. (1987) found that the topological structure of scalp voltage topographic map of resting EEG signal always remained relatively stable within a certain period of time (80–120 ms), and these topological structures were called “functional microstates.” To explore the microstates of subjects in decision task under different emotions, eeglab and Cartool software were used to calculate the ERP microstates of subjects (Hu et al., 2014). T-AAHC method was used for category recognition of microstates, and cross-validation criteria (CV) was used to confirm the selection of optimal categories (Michel and Koenig, 2018). In view of the fact that each trial relies on the first two mazes for decision-making and the duration is 3,000 and 500 ms, respectively, data from 0 to 3,500 ms of each trial are intercepted for ERP microstate analysis. Based on the above analysis, the Curve of global field power (GFP) changing with time and extracted microstates based on GFP peaks are drawn. GFP equals the root mean square (RMS) across the average-referenced electrode values at a given instant in time. It constitutes a single, reference-independent measure of response strength. Its formula is as follows:


(1) $$GFP_{u}=\sqrt{\left(\frac{1}{n}\cdot \sum_{i=1}^{n}u_{i}^{2}\right)}$$

where *u*_*i*_ refers to the voltage value of each electrode under the average voltage reference, and *n* represents the number of electrodes.

##### Effective connectivity analysis and graph theoretical analysis

Effective brain connectivity (EBC) is introduced in connectivity analysis so as to explore the causal relationship between different EEG signal channels and further detect the direction of the connection between brain regions (Nolte et al., 2008). Its basic idea mainly includes two aspects : (1) the connection of neural process needs a certain amount of time; (2) If the propagation velocity of different frequency waves is similar, the phase difference between the signal sender and receiver will increase with the increase of frequency, which is shown as a positive slope in the phase spectrum.

Based on this idea, phase slope index (PSI) is defined as follows:


(2) $${\tilde{\Psi }}_{ij}=J\left(\sum_{f\in F}K_{ij}^{*}(f)K_{ij}(f+\delta f)\right)$$

where $K_{ij}^{*}\left(f\right)$ is the coherence function, δ*f* is the frequency resolution of fast Fourier transform (FFT), *F* is the set of frequencies, and 𝔍(·) represents the imaginary part.

By calculating PSI indexes of different frequency bands (delta: 1–4 Hz, theta: 4–8 Hz, alpha: 8–13 Hz, beta: 13–30 Hz, gamma: 30–40 Hz), the directed weighted graphs between channels in different frequency bands can be obtained. To reduce spectral leakage, 50% overlapping Hamming windows are used in this paper. Considering that the small weight may be caused by the noise in the experiment process, but it may also contain valuable structural information, the optimal threshold of sparseness needs to be determined. Therefore, based on the idea of maximizing information on the networks' community structure, Bordier et al. (2017) proposed percolation analysis. The percolation threshold was defined as the threshold at which the maximum number of connected component nodes began to decrease. Through this sparsification method, we obtained a new directed weighted graph and used it for the following graph theoretical analysis.

For the description and evaluation of effective connectivity network of the brain, graph theory was integrated into the analysis of EEG (Akbarian and Erfanian, 2020). Key graph metrics describing the architecture of the network were computed from the directed and weighted adjacency matrices (PSI adjacency matrices) using BCT (Rubinov and Sporns, 2010), to include clustering coefficient (CC), local efficiency (LE), global efficiency (GE), characteristic path length (CPL). The first two metrics are local measures of the network, while the last two are the global measures of the network.

###### Global measures

Characteristic path length (CPL) is the average shortest path length of all node pairs. Its definition is as follows:


(3) $$CPL=\frac{1}{N}\sum_{i\in N}\frac{\sum_{j\in N,j\neq i}d_{ij}}{N-1}$$

where *d*_*ij*_ is the shortest path length (distance) between nodes i and j, N is the number of electrodes.

Global efficiency (GE) is the average inverse of all shortest path lengths and is used to measure the integration or overall ability to carry out parallel information transfer and fast information exchange between distributed regions. Its definition is as follows:


(4) $$GE=\frac{1}{N}\sum_{i\in N}\frac{\sum_{j\in N,j\neq i}{\left(d_{ij}\right)}^{-1}}{N-1}$$

###### Local measures

Clustering coefficient (CC) is used to measure the closeness of nodes in the network to their neighbors. Its definition is as follows:


(5) $$CC_{i}=\frac{t_{i}}{\left(k_{i}^{out}+k_{i}^{in}\right)\left(k_{i}^{out}+k_{i}^{in}-1\right)-2\sum_{j\in N}a_{ij}a_{ij}}$$

where *t*_*i*_ is the number of triangles around node *i*, $k_{i}^{in}$ and $k_{i}^{out}$ are the input and output degrees of node *i* respectively, and *a*_*ij*_ is the element of adjacency matrix.

Local efficiency (LE) measures the information transfer efficiency of a local area, which is measured by removing a node and calculating the communication efficiency between its neighbors. Its definition is as follows:


(6) $$LE_{i}=\frac{1}{2}\frac{\sum_{j,h\in N,j\neq i}\left(a_{ij}+a_{ji}\right)\left(a_{ih}+a_{hi}\right)\left[d_{jh}{\left(N_{i}\right)}^{-1}+d_{hj}{\left(N_{i}\right)}^{-1}\right]}{\left(k_{i}^{out}+k_{i}^{in}\right)\left(k_{i}^{out}+k_{i}^{in}-1\right)-2\sum_{j\in N}a_{ij}a_{ij}}$$

where *d*_*jh*_(*N*_*i*_) is the shortest path between *j* and *h* containing only neighbor *i*, and *d*_*hj*_(*N*_*i*_) similarly.

##### Statistical analysis

###### Matched samples *t*-test

For subjective data and behavioral data, matched samples *t*-test was performed on the valence scores before and after each emotional stimulus (H, Q, T) to verify the effect of emotional stimuli.

###### Analysis of variance

In order to explore whether the decision behavioral data has significant differences among different emotions, ANOVA were performed both on the decision accuracy and reaction time data of these four blocks (H, Q, T, None).

###### Independent samples0*t*-test0and pearson correlation analysis

For EEG data, in order to evaluate the relationship between the graph metrics of different subjects and their corresponding behavioral data, global measures and local measures were analyzed, respectively.

In the analysis of global measures, Pearson correlation analysis was used to explore the correlation between the global measures (GE, CPL) of different bands and the decision accuracy.

For the processing of local measures, it is firstly divided into two groups according to the decision accuracy. The 16 blocks with the highest decision accuracy are divided into a group (group1), and the 16 blocks with the lowest decision accuracy are divided into a group (group2). The whole-brain averages of the 5 bands' local measures (LE, CC) were calculated for the two groups, respectively, and the independent samples *t*-test was used to analyze whether there was a significant difference between the local measures of group1 and group2. This step is mainly to locate the bands and metrics related to the decision accuracy.

For the local measures with significant differences, the EEG topographic maps of the EEG local measures of group1 and group2 were drawn, respectively. The electrode area related to the decision accuracy was obtained by comparing between groups. Considering that the superimposed average may be affected by some subjects, the correlation between these metrics and the decision accuracy cannot be rigorously stated. As a result, the Pearson correlation analysis is used to further prove whether there is a significant correlation between the decision accuracy and the local measures of these bands.

### Results

#### The results of matched samples *t*-test showed that the three emotions were effectively stimulated

**Figure 6:**
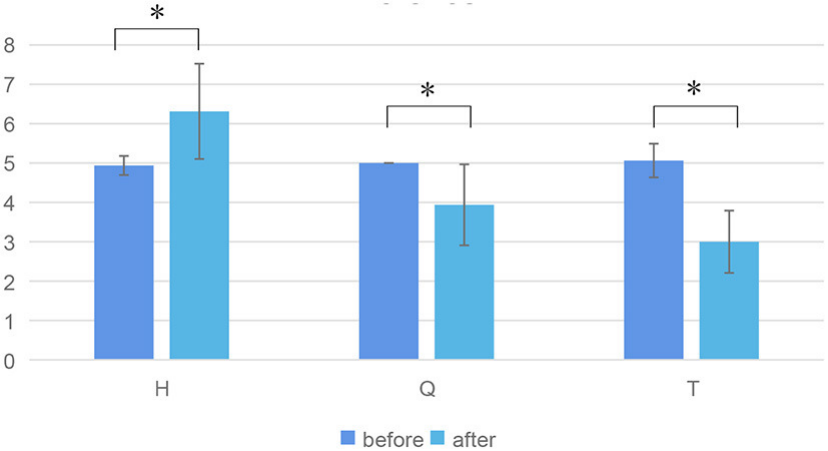
Valence score before and after three kinds of emotional stimuli. * represents significant difference between the two groups (*p* ≤ 0.05).

For hypothesis H1, the paper analyzed the subjective data (V-A scale), and the results were shown in Figure 6. Figure 6 shows the valence score before and after three kinds of emotional stimuli. Matched samples *t*-test results showed that valences were significantly different before and after each emotional stimuli (H, Q, T). Among them, the valence increased significantly stimulated by H, while the valence decreased significantly stimulated by Q and T. Therefore, it can be shown that from the perspective of continuous emotions, one positive emotion and two negative emotions are effectively stimulated.

In post-trial interviews, subjects reported that the three emotional stimuli evoked different emotions in them. The Bean clip stimulated their positive emotion. The Curve clip and Aftershock clip stimulated their negative emotions, specifically fear and sadness, respectively. This further validated that our emotional stimuli were effective.

Furthermore, ANOVA was performed on the behavioral data of decision task under different emotions. As shown in Table 1 there was no significant difference in decision accuracy and reaction time between the three kinds of emotional stimuli and no stimulus. The average correct decisions of None, H, Q and T were 32.94, 33.00, 32.75, and 32.19, respectively (totally 40), and the response time of None, H, Q and T were 495.57, 471.50, 445.93, and 460.07 ms, respectively. Although there was no significant difference in the behavioral data, we could still find that the decision accuracy was highest in the positive emotion, followed by no stimuli, and finally the two negative emotions. For reaction time, the reaction time of decision-making under fear was the shortest, and the reaction time with no stimuli was longer than that with emotional stimuli.

**Table 1:** ANOVA results for behavioral data (decision accuracy and reaction time).

		**Sum of squares**	**Mean square**	***F***	**Sig**.
Accuracy	Between groups	6.563	2.188	0.138	0.937
	Within groups	954.375	15.906		
	Total	960.938			
Reaction time	Between Groups	21348.767	7116.256	0.329	0.804
	Within groups	1254860.027	21635.518		
	Total	1276208.794			

#### Microstate analysis showed that the parietal, occipital and prefrontal lobes played key roles in spatial decision task

**Figure 7:**
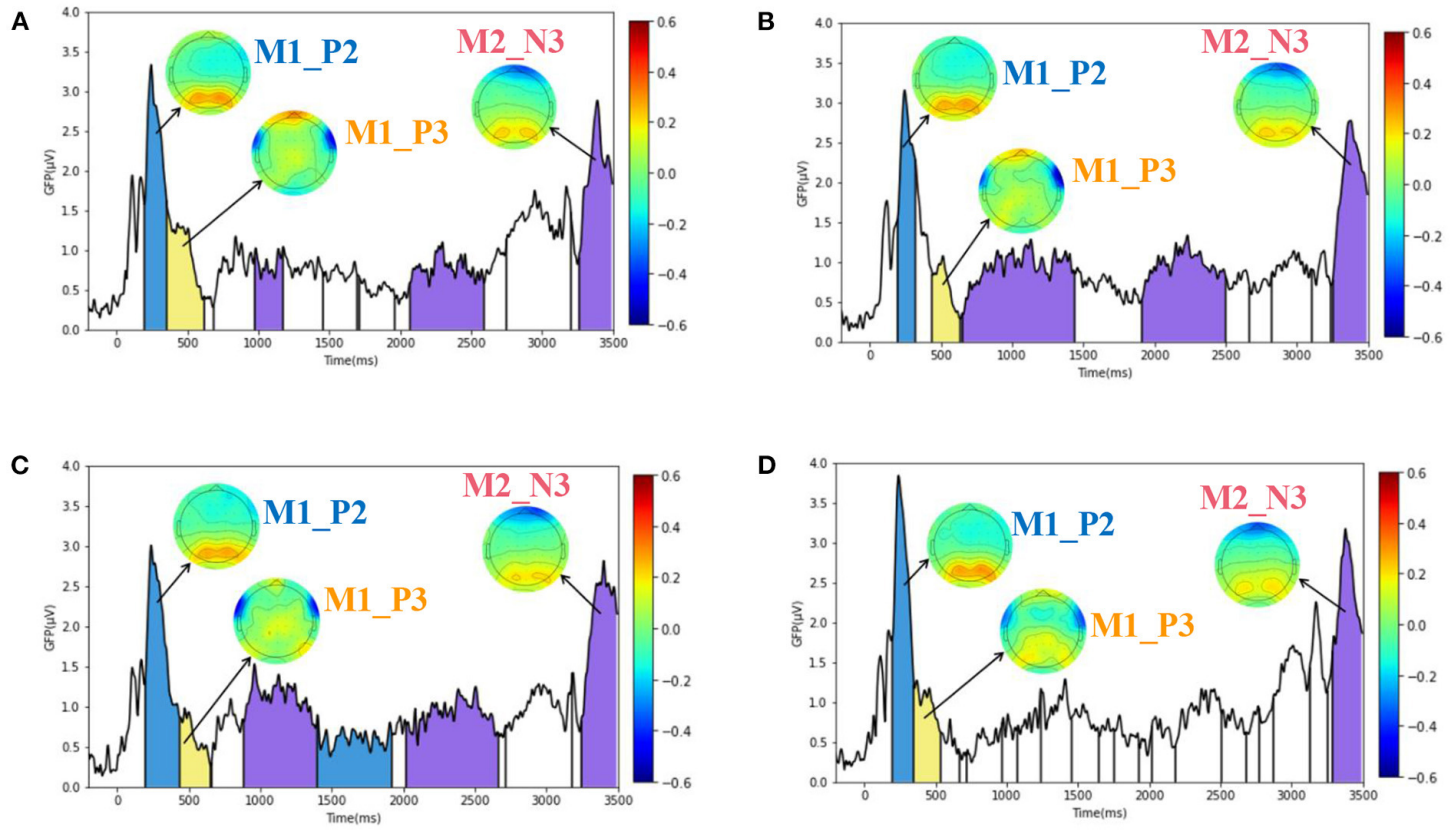
ERP microstate results of spatial decision task under four stimuli (H, Q, T, None). **(A–D)** Plotted the GFP curves and similar microstates of decision task under H, Q, T, and None emotional stimuli, respectively. There are three similar microstates, namely, M1_P2 microstate, M1_P3 microstate, and M2_N3 microstate. The corresponding time of occurrence corresponds to the blue, yellow and purple regions in each image.

For hypothesis H2, the paper explores the similar microstates of the brain in spatial decision task under different emotions through microstate analysis, and the results are shown in Figure 7. Figure 7 contains the ERP microstate analysis results of subjects in decision task under different emotional stimuli (H, Q, T) and no stimuli (None). The Y-axis in each figure is GFP value, and the X-axis is time, with a total length of 3,500 ms. Where, the first 3,000 ms corresponds to the display of Figure 1A, and the last 500 ms corresponds to the display of Figure 1B.

In the paper, the optimal number of microstates was determined by cross-validation. The number of microstates in the spatial decision task stimulated by None, H, Q, and T was 18, 10, 8, and 6, respectively. Different microstates would be separated by vertical lines in Figure 7, and microstate might appear multiple times. By comparing the similarity of microstates under four kinds of emotional state and considering the meaning of corresponding ERP components, three similar microstates were selected. The first two microstates corresponded to components P2 and P3 after the beginning of the presentation in Figure 2A, and the third microstate corresponded to components N3 after the beginning of the presentation in Figure 7B. Therefore, the three microstates were named M1_P2 microstate, M1_P3 microstate, and M2_N3 microstate, respectively. In Figure 7, similar microstates under different emotions have the same color and the same name. The blue area is M1_P2 microstate, the yellow area is M1_P3 microstate, and the purple area is M2_N3 microstate. The three microstates correspond to three EEG amplitude topographic maps in each image, respectively, and the legend is on the right side of each image.

By observing the M1_P2 microstate in Figures 7A–D, it can be found that both the parietal lobe and occipital lobe showed an obvious positive maximum value. For M1_P3 microstate, the middle prefrontal lobe showed an obvious positive maximum value. The two sides of the frontal lobe showed a negative maximum. For M2_N3 microstate, the prefrontal lobe showed a negative maximum value, while the parietal occipital lobe showed a positive maximum value. Therefore, according to the characteristics of peak appearance of microstates, it can be found that parietal lobe, occipital lobe and prefrontal lobe play a key role in spatial decision task.

Previous studies have shown that occipital lobe area is associated with vision (Pöppel et al., 1978; Berlucchi, 2014; Rehman and Al Khalili, 2019), parietal lobe area is associated with spatial positioning (Vuilleumier, 2013), and prefrontal lobe area is associated with emotion and decision-making (Manes et al., 2002; Damasio, 2006; Hiser and Koenigs, 2018). Most of these studies were based on fMRI, and some focused on patients with lesions in the prefrontal cortex (PFC). Furthermore, they paid less attention to the influence of emotions on decision-making.

Considering the role of the prefrontal lobe in emotion and decision-making, in order to further explore the influence of emotion on decision-making, we focused on the electrodes of the prefrontal lobe and used ERP to analyze how emotions affected decision-making process.

#### The amplitude difference of P2 component of prefrontal lobe revealed the influence of different emotions on the decision task

**Figure 8:**
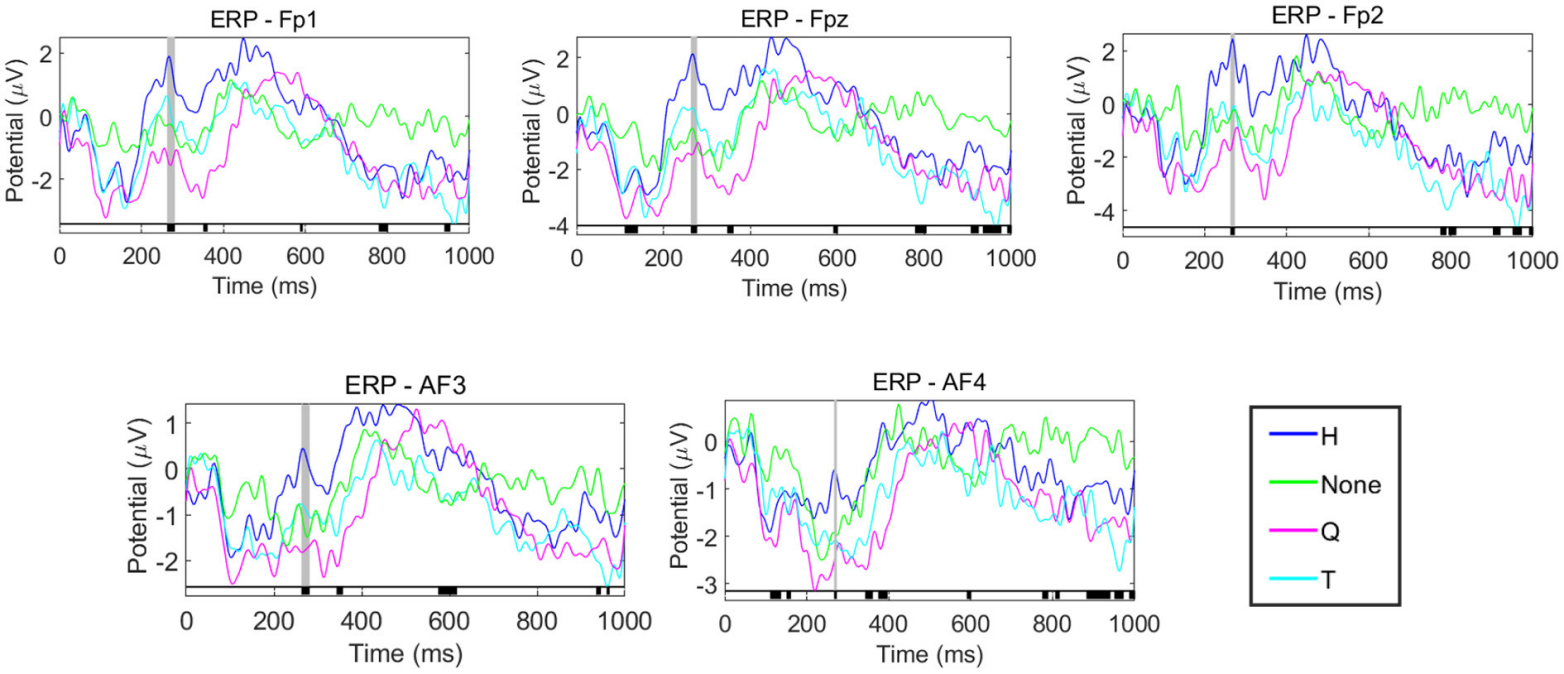
ERP analysis of prefrontal lobe electrode. From top to bottom and from left to right are the ERP waveforms of FP1, FPz, FP2, AF3, and AF4, respectively. The blue, green, purple and cyan curves represent the ERP waveforms of the decision task after the stimuli of H, None, Q, and T.

For hypothesis H3, we focused on the prefrontal lobe according to 3.2, where ERP analysis was used to explore how emotion affects spatial decision-making. ERP waveform results of prefrontal lobe under different emotional stimuli are shown in Figure 8.

Figure 8 contains the ERP waveforms of the five electrodes (FP1, FPz, FP2, AF3 and AF4) in the prefrontal lobe. The horizontal axis represents the time, the vertical axis represents the voltage value, and the four colors represent the ERP results under different stimuli. One-way ANOVA was performed for ERP data under different emotions. The periods with significant differences are shown in the black area marked on the horizontal axis in the figure. It can be seen that the four ERP curves under each electrode have significant differences in the amplitude of P2, as shown in the gray shaded area in the figure. This result indicated that different emotions would affect the P2 amplitude in the prefrontal lobe of the subjects during the decision task, thus affecting the subjects' decision-making. As shown in Figure 8, positive emotions have higher P2 amplitudes than negative emotions and no stimuli. Moreover, by comparing the P2 latency of decision task under Curve stimulus and other stimuli, it can be found that when people are nervous and afraid, they tend to have larger P2 latency and smaller P2 amplitude.

#### The decision accuracy was significantly correlated with the CC and LE of delta and theta bands in the midline region

For Hypothesis 4, this paper analyzes the graph metrics of brain network from global measures and local measures.

#### Global measures

For global measures, since the whole brain corresponds to a numerical value, a Pearson correlation analysis was performed between the global measures of the five bands and the decision accuracy. Similarly, a Pearson correlation analysis was also performed between the global measures of the five bands and the decision reaction time. The results showed that the correlation between the GE of the delta band and theta band and the decision accuracy is significant (*p* ≤ 0.01), and the correlation between the CPL of the gamma band and the decision accuracy is significant (*p* ≤ 0.01), as shown in Table 2. No significant correlation was found in the results for reaction time and global measures (*p* > 0.05).

**Table 2:** Pearson correlation analysis results between global measures and decision accuracy in five bands.

	**Delta**	**Theta**	**Alpha**	**Beta**	**Gamma**
CPL	*p* = 1.37E-1	*p* = 9.31E-1	*p* = 4.65E-1	*p* = 7.40E-2	***p*** **= 5.67E-3**
	*r* = –1.88E-1	*r* = 1.10E-2	*r* = –9.30E-2	*r* = 2.25E-1	***r*** = –3.42E-1**
GE	**p=1.68E-4**	***p*** **= 2.30E-5**	*p* = 2.55E-1	*p* = 8.31E-1	*p* = 2.25E-1
	***r*** = –4.53E-1**	***r*** = –5.03E-1**	*r* = –1.44E-1	*r* = –2.72E-2	*r* = –1.54E-1

**Means a significant correlation at the 0.01 level (two-tailed). Results with significant correlations are bolded.

#### Local measures

In the analysis of local measures, since each electrode corresponds to a numerical value, the relationship between the decision accuracy and the local measures of the brain region can be observed. Therefore, the data is first grouped by decision accuracy. Group1 is the group with higher decision accuracy, and group2 is the group with lower decision accuracy.

The results of the independent samples *t*-test between the two groups showed that only the whole-brain averaged CC and LE of delta and theta band were significantly different, whose *p*-values are shown in Table 3. Therefore, CC and LE of delta and theta bands of these two groups were selected for further detailed analysis.

**Table 3:** *T*-test analysis results of local features between group1 and group2 in five bands.

	**Delta**	**Theta**	**Alpha**	**Beta**	**Gamma**
CC	***p*** = 1.33E-2*	***p*** = 8.79E-4**	*p* = 2.11E-1	*p* = 4.78E-1	*p* = 1.56E-1
LE	***p*** = 5.23E-4**	***p*** = 3.93E-4**	*p* = 1.30E-1	*p* = 6.89E-1	*p* = 5.33E-1

*Means a significant difference at the 0.05 level (two-tailed). **Means a significant difference at the 0.01 level (two-tailed). Results with significant differences are bolded.

**Figure 9:**
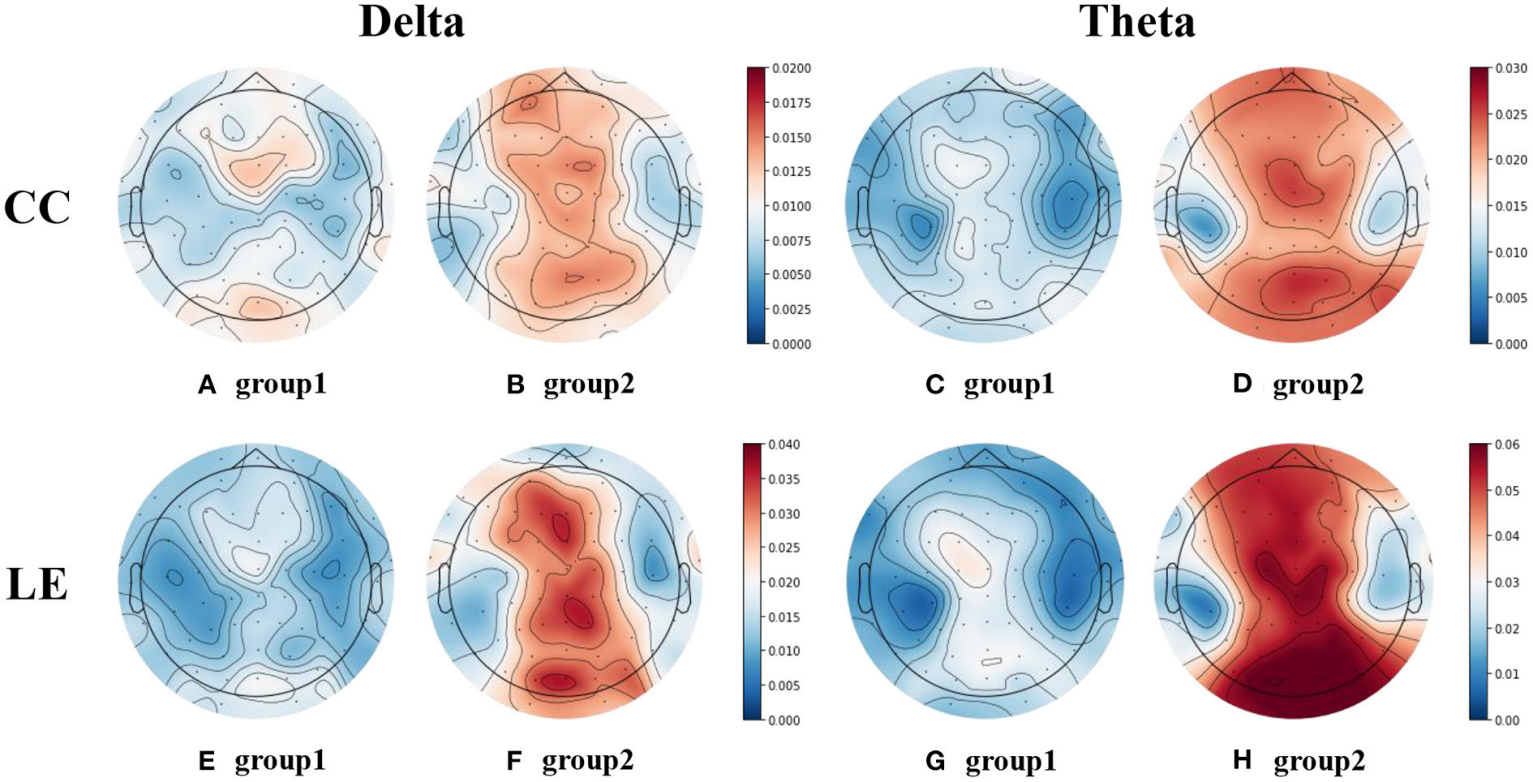
EEG topographic maps of CC and LE in delta and theta bands. **(A,B)** are the EEG topographic maps of CC in delta band for the two groups. **(C,D)** are the EEG topographic maps of CC in theta band for the two groups. **(E,F)** are the EEG topographic maps of LE in delta band for the two groups. **(G,H)** are the EEG topographic maps of LE in theta band for the two groups.

For the selected local measures, the EEG topographic maps of the two groups of them were drawn, as shown in Figure 9. Figures 9A,B represent the EEG topographic maps of the CC in delta band for group1 and group2, respectively. Similarly, (C) and (D) represent the EEG topographic maps of the CC in theta band for group1 and group2, respectively. (E) and (F) represent the EEG topographic maps of the LE in delta band for group1 and group2, respectively. (G) and (H) represent the EEG topographic maps of the LE in theta band for group1 and group2, respectively.

It can be found that the CC and LE in delta and theta bands exhibit similar patterns. In the midline region of the delta and theta bands, the CC and LE of the group with high decision accuracy were lower. We believe that there may be a relationship between the CC and LE in the midline region of these two bands and the decision accuracy.

Considering that the average EEG topograph maps of the subject data may be affected by individual subjects, it is difficult to strictly explain the negative correlation between decision accuracy and CC/LE. Therefore, the average CC in delta and theta bands of all electrodes of each subject was calculated. Pearson correlation coefficient test was carried out between the average CC in delta and theta bands and the decision accuracy data. The same analysis was performed for LE. The results showed that there was a significant negative correlation in both the delta band and theta band, as shown in Table 4. This further proves that the CC and LE in the midline region of the delta and theta bands are significantly negatively correlated with the decision accuracy of the spatial decision task.

**Table 4:** Pearson correlation analysis results between local measures and decision accuracy in delta and theta bands.

	**Delta**	**Theta**
CC	***p*** **= 4.80E-2**, ***r*** = –0.35*	***p*** **= 5.89E-5**, ***r*** = –0.65**
LE	***p*** **= 3.25E-3**, ***r*** = –0.50**	***p*** **= 2.96E-5**, ***r*** = –0.67**

*Means a significant correlation at the 0.05 level (two-tailed). **Means a significant correlation at the 0.01 level (two-tailed). Results with significant correlations are bolded.

## Discussion

### Microstate analysis showed that the parietal, occipital and prefrontal lobe played key roles in spatial decision task

In this paper, three similar microstates of decision task under different emotional stimuli are found through microstate analysis. Moreover, the prefrontal lobe, parietal lobe and occipital lobe showed obvious peaks in the three microstates, which reflected the key role of these three regions in the spatial decision-making process.

The roles of these three regions are also mentioned in the relevant studies. Si et al. pointed out in their study that decision-making was thought to involve the activation of distributed cortical regions, such as frontal, parietal, and occipital from the results of scalp topology and cortical activation (Si et al., 2021). Some studies further confirm the reliability and validity of the three key regions derived from our study and can further help explain what role these regions play in spatial decision-making. Regarding the role of the occipital lobe area, a large number of research has confirmed the correlation between the occipital lobe area and vision (Pöppel et al., 1978; Berlucchi, 2014; Rehman and Al Khalili, 2019). For the role of the prefrontal and parietal lobes, Patai et al. combined evidence from non-spatial studies and found that the interaction between the lateral PFC and the posterior parietal cortex is important for planning paths and processing subgoals (Patai and Spiers, 2021). Existing research suggests that the PFC supports decision-making, target tracking, and planning. And PFC plays an important role in spatial navigation, supporting flexible response to environmental changes (Javadi et al., 2019). By summarizing 47 fMRI works on spatial navigation, Li et al. (2021) pointed out that the brain regions involved in human spatial navigation are mainly located in the medial temporal cortex and posterior parietal cortex, and different spatial scales and spatial reference frames may show different brain activation patterns. Similarly, studies have shown that information about correct decisions are stored in the ventral temporal cortex and posterior parietal cortex (Philiastides et al., 2010; Hutchinson et al., 2015), while the ventromedial prefrontal cortex (vmPFC) is implicated in computing expected value and reward outcomes in processing decisions (Daw and Doya, 2006).

For the M1_P2 microstate, there were peaks of voltage in the parietal and occipital lobes. Kravitz et al. (2011) pointed out that the role of the occipital-parietal pathway is mainly to integrate and abstract visual information from the retina. Therefore, this microstate may correspond to the process of human beings receiving and processing visual information. For the M1_P3 microstate, the corresponding prefrontal lobe showed a peak. Considering the peak in the parietal lobe area of the previous microstate, and the parietal-prefrontal pathway is mainly used to control eye movements and participate in spatial attention activities, this microstate may correspond to the decision-making process of the subjects. For the M2_N3 microstate, peaks were seen in the prefrontal, parietal and occipital lobes. The activation of occipital and frontal was thought to involve in the identification of options and provide information for humans to sift through (Si et al., 2019). Considering that this microstate occurs after the appearance of the second maze (Figure 2B), it is necessary to integrate the visual information of the second maze and call the spatial navigation ability to make decisions, it is likely that this state reflects multiple areas collaborate to make decisions.

### The amplitude difference of P2 component of prefrontal lobe revealed the influence of emotion on the decision task

Emotion-related studies have shown that the PFC plays a key role in the production and regulation of emotion (Dixon et al., 2017). Several ERP studies have demonstrated a correlation between P2 components and emotions (Ma et al., 2015; Wang et al., 2019a). Under different emotions, the corresponding P2 components of the subjects are often different. The study by Spreckelmeyer et al. (2006) showed that positive emotions evoked larger P2 amplitudes than neutral and negative emotions.

In this paper, the ERP waveforms of subjects during decision task under different emotional stimuli in the prefrontal lobe were plotted. The results also showed that subjects with positive emotion produced the largest P2 amplitudes during the decision task compared to negative emotions and no stimuli (see Figure 8). Different from the study by Spreckelmeyer et al., which used the data in the process of emotional stimuli for analysis, this paper uses the decision data after different emotional stimuli for analysis. On the one hand, we further confirmed the effect of different emotions on the P2 component and the persistent role of emotion in decision task. On the other hand, this also indicates that the influence of emotion on decision-making may be reflected in the difference of P2 components.

### The decision accuracy was significantly correlated with the CC and LE of delta and theta bands in the midline region

In terms of global measures, the results of the effective connectivity analysis combined with the graph theoretical analysis showed that, there was a significant negative correlation between the decision accuracy and the GE of the delta band and theta band. Similarly, there was also a significant negative correlation between the decision accuracy and the CPL of the gamma band. By exploring brain connectivity in children with attention deficit and hyperactivity disorder (ADHD), Furlong et al. found that increased GE was associated with increased severity of inattention symptoms (Furlong et al., 2021). Therefore, it was speculated that compared with the group with higher decision accuracy, the group with lower decision accuracy may be distracted, and the decision-making results were relatively poor. This distraction manifested itself in the lower CPL of the gamma band and the lower GE of the delta and theta bands.

In terms of local measures, the decision accuracy had a significant negative correlation with the CC and LE of the delta and theta bands in the midline region. Existing studies have analyzed the associations between delta band, theta band, and midline regions with decision-making and cognitive processes, and have drawn some conclusions that can be used to explain the results of our analysis. Studies by Li et al. (2010), Jensen and Tesche (2002), and Massar et al. (2014) indicated that theta oscillations (4-8 Hz) in the frontal lobe are associated with different aspects of human behavior (risk decision-making and risk learning). Cohen et al. (2007) experimentally found that EEG responses to losses, compared to wins, were associated with enhanced power and phase coherence in the theta frequency band. Pinner and Cavanagh (2017) also pointed out that the frontal midline theta is not only a candidate mechanism for implementing cognitive control, but is also sensitive to the inherent costs therein. Wang et al. studied human brain activity during reinforcement learning and demonstrated that theta and delta oscillations reflect separable components of higher-level cue processing prior to decision feedback. Feedback unavailable cues were more likely to induce an increase in mid-posterior delta power than feedback available cues after a decision (Wang et al., 2016). Therefore, it is speculated that compared with the group with higher decision accuracy, the group with lower decision accuracy may be more sensitive to the loss in decision task. The subjects' higher sensitivity to loss feedback may be reflected by higher CC and LE of the delta and theta bands in the midline region, which means subjects are more anxiety about loss.

In summary, the results of the global measures and local measures suggest that the group with lower decision accuracy may reduce the attention of the decision task itself and be more sensitive to loss feedback, which in turn affects the decision-making results.

## Conclusion and future work

In this paper, we designed a novel experimental task to explore the influence of emotions on spatial decision-making. During the experiment, we collected the subjective and objective data of the subjects. The subjective data verified the emotions were effectively stimulated. For the objective data, three conclusions were drawn. Firstly, we observed three similiar ERP microstates in the decision-making process under different emotions by microstate analysis. Additionally, the prefrontal, parietal and occipital lobes played key roles in decision task. Secondly, we found that the P2 component of the prefrontal lobe represents the influence of different emotions on decision-making by ERP analysis. Among them, positive emotion evoked the largest P2 amplitude compared to negative emotions and no stimuli. Finally, we explores graph metrics of brain network related to decision accuracy. In terms of global measure, there is a significant negative correlation between the decision accuracy and the GE of the delta and theta bands, and CPL of the gamma band. In terms of local measures, the decision accuracy has a significant negative correlation with the CC and LE of the delta and theta bands, and this effect is especially reflected in the midline region.

Although this paper obtained some useful conclusions, it still needs further improvement and refinement. Three key regions (the prefrontal, parietal and occipital lobes) are found in spatial decision task, but the role of these three regions in the spatial decision task needs to be explored in more detail. Positive emotion evokes the largest P2 amplitude compared to negative emotions and no stimuli in spatial decision task, but whether this conclusion is also applicable to other decision tasks requires further analysis and exploration. Because of MFN and FRN was strongly correlated with decision-making (Garrido-Chaves et al., 2021; Si et al., 2021), the following research will further analyze the difference of ERP components such as MFN and FRN in decision-making under different emotions. Finally, the metrics that significantly correlated with decision accuracy can be further used to predict people's decision-making, and it needs us to establish classification model for further analysis.

## Data availability statement

The raw data supporting the conclusions of this article will be made available by the authors, without undue reservation.

## Ethics statement

The studies involving human participants were reviewed and approved by Casia Human Subjects Research Application. The patients/participants provided their written informed consent to participate in this study. Written informed consent was obtained from the individual(s) for the publication of any potentially identifiable images or data included in this article.

## Author contributions

YZ, DW, XW, and SC contributed to conception and design of the study. YZ organized the database, performed the statistical analysis, and wrote the first draft of the manuscript. All authors contributed to manuscript revision, read, and approved the submitted version.

## Funding

This research is supported by the National Natural Science Foundation of China under Grant Nos. 61872363 and 61672507, the Natural Science Foundation of Beijing and Key project of Science and Technology Plan of Beijing Municipal Education Commission under Grant No. 21JD0044, the Strategic Priority Research Program of Chinese Academy of Sciences, Grant No. XDA27000000, the National Key Research and Development Program under Grant No. 2016YFB0401202, and the Research and Development Foundation of The Institute of Automation, Chinese Academy of Sciences under Grant No. Y9J2FZ0801.

## Conflict of interest

The authors declare that the research was conducted in the absence of any commercial or financial relationships that could be construed as a potential conflict of interest.

## Publisher's note

All claims expressed in this article are solely those of the authors and do not necessarily represent those of their affiliated organizations, or those of the publisher, the editors and the reviewers. Any product that may be evaluated in this article, or claim that may be made by its manufacturer, is not guaranteed or endorsed by the publisher.
